# Conformation-Specific Inhibitory Anti-MMP-7 Monoclonal Antibody Sensitizes Pancreatic Ductal Adenocarcinoma Cells to Chemotherapeutic Cell Kill

**DOI:** 10.3390/cancers13071679

**Published:** 2021-04-02

**Authors:** Vishnu Mohan, Jean P. Gaffney, Inna Solomonov, Maxim Levin, Mordehay Klepfish, Sophia Akbareian, Barbara Grünwald, Orly Dym, Miriam Eisenstein, Kenneth H. Yu, David P. Kelsen, Achim Krüger, Dylan R. Edwards, Irit Sagi

**Affiliations:** 1 Department of Biological Regulation, Weizmann Institute of Science, Rehovot IL76100, Israel; vishnu.mohan@weizmann.ac.il (V.M.); jean.gaffney@baruch.cuny.edu (J.P.G.); inna.solomonov@weizmann.ac.il (I.S.); maxim298@gmail.com (M.L.); moty.kl@gmail.com (M.K.); 2 School of Biological Sciences, University of East Anglia, Norwich NR4 7TJ, UK; sophieaelias@outlook.com (S.A.); dylan.edwards@uea.ac.uk (D.R.E.); 3 Institut für Molekulare Immunologie und Experimentelle Onkologie, Klinikum Rechts der Isar der Technischen Universität München, 81675 Munich, Germany; barbara.gruenwald@googlemail.com (B.G.); achim.krueger@tum.de (A.K.); 4 Structural Proteomics Unit, Faculty of Biochemistry, Weizmann Institute of Science, Rehovot IL76100, Israel; Orly.Dym@weizmann.ac.il; 5 Department of Molecular Genetics, Faculty of Biochemistry, Weizmann Institute of Science, Rehovot IL76100, Israel; miriam.eisenstein@weizmann.ac.il; 6 Department of Medicine, Memorial Sloan Kettering Cancer, New York, NY 10065, USA; yuk1@mskcc.org (K.H.Y.); kelsend@mskcc.org (D.P.K.)

**Keywords:** matrix metalloproteinase-7, matrilysin, monoclonal antibody, pancreatic ductal adenocarcinoma, fas ligand, gemcitabine, oxaliplatin, drug synergy

## Abstract

**Simple Summary:**

Extracellular matrix remodeling enzymes are dysregulated in several pathologies. Our aim was to generate a unique function blocking monoclonal antibody against one such cancer-associated enzyme, matrix metalloproteinase 7 (MMP-7). We generated GSM-192, with high affinity and specificity towards active MMP-7, utilizing a sequential immunization strategy. GSM-192 induced pancreatic cancer cell apoptosis, reduced migration and increased sensitivity to chemotherapeutics. Our study highlights the use of GSM-192 as a valuable therapeutic, diagnostic and research tool.

**Abstract:**

Matrix metalloproteases (MMPs) undergo post-translational modifications including pro-domain shedding. The activated forms of these enzymes are effective drug targets, but generating potent biological inhibitors against them remains challenging. We report the generation of anti-MMP-7 inhibitory monoclonal antibody (GSM-192), using an alternating immunization strategy with an active site mimicry antigen and the activated enzyme. Our protocol yielded highly selective anti-MMP-7 monoclonal antibody, which specifically inhibits MMP-7′s enzyme activity with high affinity (IC_50_ = 132 ± 10 nM). The atomic model of the MMP-7-GSM-192 Fab complex exhibited antibody binding to unique epitopes at the rim of the enzyme active site, sterically preventing entry of substrates into the catalytic cleft. In human PDAC biopsies, tissue staining with GSM-192 showed characteristic spatial distribution of activated MMP-7. Treatment with GSM-192 in vitro induced apoptosis via stabilization of cell surface Fas ligand and retarded cell migration. Co-treatment with GSM-192 and chemotherapeutics, gemcitabine and oxaliplatin elicited a synergistic effect. Our data illustrate the advantage of precisely targeting catalytic MMP-7 mediated disease specific activity.

## 1. Introduction

Proteases are thought to represent 5 to 10% of probable drug targets [1], and the matrix enzymes of the MMP or the ADAM family of proteases, are prominent among them. MMPs and ADAMs have a heightened activity profile in tissues or cellular microenvironments that are undergoing remodeling or repair, and in those that are inflamed or diseased. Several advanced phase clinical trials using second-generation small molecule MMP inhibitors (MMPIs) failed to demonstrate efficacy [2]. However, emerging credible drug target validation, combined with increased knowledge about MMP substrates, and the physiological functions of MMPs at various stages of cancer development, offer ways around this temporary setback. It is now understood that true targets should not be compensated by changes in other pathway mediators [2], and have to be involved in facilitating passage through crucial disease checkpoints. The catalytic domains of all MMPs show similarity in their amino acid sequences, and their active site sequences are highly conserved. Hence, these active sites are notoriously difficult to target selectively using small molecules. Thus, there is a compelling case for the development of new, highly selective and potent function blocking inhibitors, such as monoclonal antibodies (mAbs) against more MMPs and ADAMs [3,4]. The active site of MMPs, a metal–protein catalytic complex, is functionally essential, and is unlikely to develop drug resistance through genetic or epigenetic alternations. Yet, achieving the ultimate goal of superior potency and selectivity of Ab will require it to bind additional enzyme surface epitopes.

Among MMPs, matrix metalloproteinase 7 (MMP-7, matrilysin) has emerged as an important target for development of next generation cancer therapeutics, due to its association with the clinical behavior of multiple tumor types. MMP-7 null mouse is prone to loss of metaplastic lesions following pancreatic ductal ligation and MMP-7 is important for acinar to ductal metaplasia in PDAC [5,6], and inhibition of MMP-7 with a broad spectrum MMP inhibitor reduced the number of intestinal polyps [7].

Notably, MMP-7 is the simplest MMP, as it lacks a hemopexin domain and is made of only the propeptide (in the zymogen form) and catalytic domains [8]. Nevertheless, mounting evidence indicates that it plays an important role in ECM remodeling, innate immunity in organs [9] and inflammation [10]. MMP-7 is responsible for the breakdown of macromolecules like type IV collagen, gelatins, laminin, entactin/nidogen, and tenascin-C [11]. In addition to these classical enzymatic roles, MMP-7 has been implicated in modifying signaling pathways and regulating activity of cytokines [12]. In the non-canonical signaling-related activity of MMP-7, the best-characterized substrates include Fas-L, Fas-R/CD-95 [13], TNF-α, VEGF [14], plasminogen [15], E-cadherin [16], and integrin β-4 [17]. MMP-7 is involved in regulation of ErbB4 activity [18], induction of IL-17 mediated epithelial to mesenchymal transition [19], and acts as pro-invasive effector molecule via the Wnt/β-catenin pathway [20]. Through these effects, MMP-7 acts on tumor cells as well as on stromal cells, with multiple downstream implications in both the protease web and signaling cascades. Remarkably, MMP-7 processing of the Fas/FasL system is implicated in the acquisition of drug resistance against the chemotherapy drug oxaliplatin, in colon cancer cells [21]. Furthermore, a high expression of MMP-7 was also found in patients with colorectal cancer undergoing 5-fluorouracil-based adjuvant chemotherapy [22].

Considering the numerous deleterious activities of active MMP-7 in diseased tissue, there is great potential in specifically targeting active MMP-7. Here, we expanded on our previous approach for production of function blocking mAbs targeting MMP active conformation, by using a sequential immunization strategy of both synthetic and recombinant enzyme epitopes. The resultant anti MMP-7 mAb GSM-192 induced pancreatic cancer cell apoptosis, reduced migration and improved efficacy of chemotherapeutics. GSM-192 is a valuable therapeutic, diagnostic and research tool to validate MMP-7 as a suitable drug target in cancer and inflammation.

## 2. Materials and Methods

### 2.1. Antibody Generation and Purification

Female BALB/c mice were immunized with complete Freund’s adjuvant (Disco) and 30 μg of the catalytic domain of MMP-7 or Zn Tripod-KLH, and boosted every 2 weeks with incomplete Freund’s adjuvant by subcutaneous injection. Mouse bleeds were screened for reactivity to antigens in ELISA performed with singletons from limited volume mouse bleed. Spleens from selected mice were collected, and B cells were fused with NSO murine myeloma cells. Hybridomas were screened with ELISA, and selected hybridomas were subcloned and expanded in tissue culture. Hybridoma cells of GSM-192 were grown in DCCM (serum-free medium designed for hybridoma cell growth and monoclonal antibody production, purchased from Biological Industries, Beit-Haemek, Israel). Cells were precipitated by centrifugation at 193× *g*, and the supernatant was collected. The supernatant was dialyzed against 20 mM phosphate buffer (pH 8). A 1 mLHiTrap protein A high-performance column was equilibrated with 100 mM phosphate buffer (pH 8), and the supernatant was loaded at 1 mL/min. The antibody was eluted with 100 mM citrate buffer (pH 6) and dialyzed against 50 mM Tris (pH 7.5) and 150 mM NaCl.

### 2.2. Antibody Digestion with Papain for Fab Generation

Papain was activated in 0.5 M Tris-HCl (pH 8), 10 mM EDTA, and 5 mM dithiothreitol for 15 min at 37 °C. Active papain was added to a solution of intact GSM-192 at a ratio of 1:1000, and the digestion process was carried out for 3 h at 37 °C. The digestion reaction was terminated with the addition of 20 mM iodoacetamide in the dark at room temperature for 30 min. The Fab fragment was isolated from the Fc by a protein A column, and the Fab fragment was collected from the flow- through and dialyzed against 50 mM Tris (pH 7.5) and 150 mM NaCl. The purity of the Fab fragment was estimated at ~95% by 12% SDS-PAGE gel.

### 2.3. MMP-7 Elisa Binding Assay

A 96-well plate (Nunc) was coated with MMPs at 5 mg/mL. After coating, the plate was incubated with the GSM-192 at 25 °C for 1 h. The bound Fab fragment/full length was detected using a goat anti-Fab/anti-mouse antibody followed by peroxidase-conjugated bovine anti-goat antibody according to standard procedures. A four-parametric sigmoidal curve-fitting analysis was used to calculate the half-maximal effective concentration.

### 2.4. MMP-7 Enzymatic Kinetic Assay

The enzymatic activity of MMPs in the presence of GSM-192 Fab was measured at 37 °C by monitoring the hydrolysis of fluorogenic peptide Mca-Pro-Leu-Gly-Leu-Dpa-Ala-Arg-NH2 at λex = 340 nm and GSM-192 at λex = 390 nm as described previously [23]. A range of different concentrations of GSM-192 Fab (0–200 nM) were incubated with 10 nM of MMPs in 50 mM Tris-HCl buffer (pH 7.5 at 37 °C), 100 mM NaCl, 5 mM CaCl_2_, and 0.05% Brij-35 for 40 min at 37 °C. The enzymatic reaction was initiated by addition of the fluorogenic peptide to a final concentration of 10 μM. Fluorescence was recorded immediately and continuously for 30 min. Initial reaction rates were measured, and the inhibition constant was evaluated by fitting the data to the equation below where V_i_ is the initial velocity in the presence of the inhibitor, V_0_ is the initial velocity in the absence of the inhibitor, and I is the inhibitor concentration.

$$\frac{\mathrm{Vi}}{\mathrm{Vo}}\%=\frac{1}{1+\frac{\left[I\right]}{\mathrm{IC}50}}\;\times \;100$$

### 2.5. Antibody Sequencing

Immunoglobulin V region genes were cloned and sequenced after amplification by PCR. The total RNA was prepared from 5 × 10^6^ hybridoma cells by the phenol-guanidine isothiocyanate method (peqGOLD TriFast of peqlab biotechnologie), according to the manufacturer’s protocol. CDNA was obtained, and amplification was performed in one step, using Reverse-iTTM one step RT-PCR Kit (ABgene). V region genes were amplified by using degenerate sense primers, homologous to the mouse heavy and light chain leader sequences and antisense constant primers (Amersham Biosciences). The amplification products were ligated into the pGEM-T Easy Vector (Promega) by using standard protocols, and both strands of inserts were sequenced on an automated sequencer at the DNA sequencing unit (Biological Services, Weizmann Institute of Science).

### 2.6. Protein Crystallography of GSM-192 Fab

The crystals of free GSM-192 Fab, obtained using the hanging drop vapor diffusion method diffracted at best to a 2.3 Å resolution. The crystals were grown from 200 mM CaCl_2_, 100 mM HEPES Ph = 7, 20% PEG 6000, and 10 mM betaine hydrochloride. The crystals formed in the trigonal space group P3121, with cell constants a = 88.44, b = 88.44, and c = 119.34 Å and g = 120° with one copy of the light and heavy chains. A full X-ray data set to 2.3 Å resolution was collected at 100 K using an RU-H3R rotating anode with an RAXIS IV++ detector. The diffraction images were indexed and integrated using the HKL2000 program [24]. Processing of X-ray diffraction data collected in oscillation mode and the integrated reflections was scaled using the SCALEPACK program [24,25]. Structure factor amplitudes were calculated using TRUNCATE from the CCP4 program suite [26]. Details of the data collection are described in Table S1. The structure was solved by molecular replacement with the PHASER program [27], using the refined structure of the Anti-Abeta monoclonal antibodies mAbs (PFA1) (Protein Data Bank (PDB) accession code 2IPU) as a model. All steps of atomic refinements were carried out with the PHENIX program [28]. The model was built into 2mFobs-DFcalc, and mFobs-DFcalc maps by using the COOT program [29]. Refinement weights were optimized. The GSM-192 Fab construct is composed of 224 amino acid residues in the heavy chain and 215 amino acids in the light chain (439 residues). The final model includes residues 1–221 in the heavy chain and 2–185 in the light chain. The R*free* value is 27.85% (for the 5% of reflections not used in the refinement), and the R*work* value is 23.22% for all data to 2.3 Å. The GSM-192 Fab model was evaluated with the PROCHECK program [30]. Details of the refinement statistics of the GSM-192 Fab structure are described in Table S1. The coordinates and structure factors for GSM-192 Fab have been deposited in the PDB under the ID code 6FBJ.

### 2.7. Computational Modeling and Docking

The Fv domains of antibody GSM-192 were computationally docked to MMP-7. Comparison of the several structures of MMP-7 available in the PDB showed variations in the structure, which affected the width of the active site cleft. Normal modes analysis [31], applied to the experimental structures, showed similar mobility of the loops. Therefore, the experimental structures and several normal modes conformers of MMP-7 were used in docking.

The molecules were docked using the FFT-based geometric-electrostatic-hydrophobic (GEH) version of MolFit [32,33,34], which executes an exhaustive step-wise scan of the relative rotations and translations of the docked molecules, and provides a GEH score for every tested position. The resultant poses were filtered using a post-scan propensity and solvation (P&S) filter [35]. The filtered models were further screened to include only models where the interaction involves exposed residues in the antibody CDRs. This screen counted the number of atom–atom contacts (≤5 Å distance) between exposed CDR residues and the target molecule.

The GEH score of MolFit is sensitive to small changes in the relative orientation of the molecules [36], and local rigid-body refinements were previously found to be very effective for identifying genuinely high-scoring docking models. Therefore, the models from the several scans were refined, by allowing small local rotations in steps of 2°. The refinement highlighted one model in the docking results. This model was ranked 1 in the docking scan that employed a normal modes conformer closely resembling structure 2y6a, and its refined score was 3.1σ above the next model and 9σ above the mean score (mean score and σ were determined by fitting an extreme value distribution function to the distribution of GEH scores [36]). Notably, the same model was obtained in scans that included AHA as part of the MMP-7 structure and scans without AHA. In the latter case, the location of AHA was empty and accessible.

Anchoring spots were used to identify preferred binding locations of single amino acid side chains on the surface of proteins. The mapping was performed with ANCHORSmap [37].

We used UCSF-Chimera [38] for structure analyses and comparisons.

### 2.8. Constructing and Analyzing, MMP Ortholog-Based Multiple Sequence Alignment (MSA)

To construct multiple sequence alignment, MMP protein sequences were aligned using the Geneious 7.1.9 (https://www.geneious.com, accessed on 13 December 2020) software selecting ClustalW [39] with its default parameters. Annotated (Swiss-Prot) MMP protein sequences were downloaded from UniProt (https://www.uniprot.org/, accessed on 13 December 2020) [40], selecting five closely related species (from family Mammalia, and specifically clade Eutheria): *Homo sapiens, Mus musculus, Rattus norvegicus, Sus scrofa and, Bos taurus*. This was followed by manual screening to eliminate sequences that lacked main MMP features (e.g., highly conserved HExGHxxGxxH motif), reaching a final list of 94 sequences that were organized by ortholog groups. Phylogenetic tree was constructed based on the aforementioned MSA using the “Geneious Tree Builder” tool, following the Jukes-Cantor model, using the neighbor-joining method, and resampling the tree by the Bootstrap method under default parameters.

### 2.9. Structural Alignment and Analysis

PyMol software (The PyMOL Molecular Graphics System, Version 2.0 Schrödinger, LLC.) was used to visualize and superimpose the docking model. Specifically, hMMP7 structure was super imposed with mMMP7 (SWISS-MODEL default parameters, based on UniProtKB-Q10738, Y95-Y258, using 2y6c.1.A as template) and with the closest available structure that appeared applying the NCBI BLASTP search tool (https://blast.ncbi.nlm.nih.gov/Blast.cgi?PAGE=Proteins, accessed on 13 December 2020) [41] confined to human sequence and PDB database, i.e., PDB-Id: 1USN (hMMP3).

### 2.10. Cell Culture

Pancreatic ductal cancer cell lines AsPc-1, BxPC-3, CFPAC-1 were obtained from the Ravid Straussaman lab (Weizmann Institute of Science, Rehovot, Israel). Su86.86 cell line was obtained from the Dylan Edwards laboratory, University of East Anglia. All cell lines were kept in a humidified incubator at 37 °C with 5% CO_2,_ and cultured in complete media consisting of DMEM, RPMI medium 1640, or IMDM supplemented with 10% fetal calf serum, 1% penicillin/streptomycin, 1% L-glutamine, 1% sodium pyruvate.

### 2.11. MMP-7 Lentiviral Silencing and FACS Analysis

Stable knockdown of secreted MMP-7 in AsPC-1 pancreatic cancer cells was achieved by HIV-based stable transduction with shRNA against MMP-7 using the ViraPower Lentiviral Expression System (Thermo Fisher Scientific, Waltham, MA, USA) in combination with commercially available shRNA oligos (Sigma Aldrich, St. Louis, MO, USA). Recombinant retrovirions were generated according to standard protocols [42]. AsPC-1 cell were transduced at an MOI, of 10 in the presence of 8 µg/mL polybrene. In 48 h post transduction, AsPC-1 cell were passaged and allowed to adhere for 6 h before 40 µg/mL puromycin was added to positively select transduced cells. Antibiotic pressure was maintained until 24 h after 100% of non-transduced control cells maintained at identical conditions were killed. Knockdown efficacy was determined by Western blot. Cell cycle analysis using propidium iodide in FACS was performed on the stable knockdown cells maintained at 80% confluency.

### 2.12. MTT Cell Proliferation Assay

MTT solutions (M5655 SIGMA) was added at 10% of total culture volume (0.5 mg/mL MTT in RPMI/IMDM without phenol red) and after 3 h incubation the plate was washed with PBS and solubilized using MTT solvent (0.1 N HCl in anhydrous isopropanol). The read out was obtained at 630–690 nm wavelengths in the spectrophotometer plate reader. Relative percentage of cell proliferation was determined in comparison to the control wells. A curve fitting analysis for IC_50_ was performed using Origin software v8.5.

### 2.13. FACS Annexin-V Apoptosis Detection Assay

In this assay, 70% confluent cells in 6 cm plates were treated with sterile GSM-192 Fab fragment/control anti LOXL-2 Fab fragment for 24 h and prepared for FACS using the Annexin-V FITC apoptosis detection kit (E Biosciences 88-8005-72) as described previously [43]. The FACS acquisition was performed using FACS LSR-II machine in Cell Quant software (Becton Dickinson), and further analysis was completed on FlowJo software (Treestar, Inc., San Carlos, CA, USA).

### 2.14. Western Blots

Treated cells were lysed with RIPA buffer containing protease inhibitors, and shaken intermittently while stored in ice for 30 min. The debris was removed by centrifugation at 14,000× *g* and supernatant was used for BCA protein determination assay. Alternatively, culture supernatants were concentrated at least 10 times using 0.2 μm centricons. Once normalized to equal protein content, sample buffer was added and heated for 3 min at 95 °C. The denatured samples were centrifuged for 5 min at 14,000× *g* before 25 μL was loaded onto 12% SDS page gel and standard blotting procedures were followed (General Protocol for Western Blotting, BioRad bulletin 6376). Primary Abs used were MMP-7 (Abcam, ab5706), FasL (Abcam, ab15285), α Tubulin Ab (SC31779, Santa Cruz Biotechnology, Inc., Dallas, TX, USA), and vinculin (V4505, Sigma Aldrich, St. Louis, MO, USA).

### 2.15. Wound Healing Assay (Scratch Assay)

The migratory behavior of CFPAC-1 cells was assayed by means of a wound-healing assay using a culture-insert (ibidi GmbH, Am Klopferspitz 19, Martinsried, Germany) according to the manufacturer’s instruction. After 24 h, the culture-inserts were removed leaving a cell-free gap (“defined as a wound”) of approx. 500 µm. The migration rate into this “wound area” was documented and measured using DeltaVision Microscopy Imaging Systems(GE Healthcare Life Sciences, Piscataway, NJ, USA). The migration rate was determined by the percentage area covered by cells. Each analysis was performed in triplicates.

### 2.16. Trans-Well Migration Assay

CFPAC-1 cells were starved overnight in assay media (IMDM media containing only 1% serum). Cells (1 × 10^5^) were added to the top chambers of 24-well transwell plates (BD Biosciences; 8 μm pore size), and assay media, with and without GSM-192, was added to both chambers. After overnight incubation, cells that failed to migrate on the top well were removed, and the cells that migrated to the bottom were fixed with 4% PFA and stained with crystal violet (0.05%). The numbers of cells in five fields were counted under 20× magnification, and the mean for each chamber was determined.

### 2.17. Immunohistochemistry

The study was conducted according to the guidelines of the Declaration of Helsinki, and approved by the Institutional Review Board (or Ethics Committee) of Memorial Sloan-Kettering Cancer Center (MSKCC IRB 06-107). Frozen human PDAC sections in OCT (10 µm thick) were cut on a cryostat. Sections were dried on bench top for 30 min at room temperature, rehydrated in PBS for 10 min, pre-incubated with PNB blocking buffer (PerkinElmer Life Sciences) for 1 hat room temperature, and then incubated with the primary antibody of interest, GSM-192 mAb or MMP-7 (abcam, ab5706) overnight at 4 °C. The corresponding secondary HRP antibodies were used at manufacturer’s recommended dilutions. The slides were mounted on a mounting medium. The images of mounted sections were obtained using Carl Zeiss optical microscope.

### 2.18. Co-Treatment with Chemotherapeutics

A total of 10,000 AsPC-1 or BxPC-3 cells per well were seeded into a 96 well flat bottom plate and incubated overnight in a humidified incubator at 37 °C with 5% CO_2_. Media in wells were replaced by RPMI medium with 10% FCS containing GSM-192 (2.3 µM for AsPC1 or 1.5 µM for BxPC-3) or PBS. The wells were treated with increasing concentrations of drugs (AsPC-1; 0–200 µM Gemcitabine, 0–225 µM Oxaliplatin, and BxPC-3; 0–50 µM Gemcitabine, 0–100 µM Oxaliplatin) and allowed to incubate for 72 h. The plates were further incubated for 72 h in a humidified incubator at 37 °C with 5% CO_2_. MTT solutions (M5655 SIGMA) were added at 10% of total culture volume (0.5 mg/mL MTT in RPMI/IMDM without phenol red) and after 3 h incubation, the plate was washed with PBS and solubilized using MTT solvent (0.1 N HCl in anhydrous isopropanol). The read out was obtained at 650 nm wavelength in the spectrophotometer plate reader and the background read out was performed at 570 nm. Cell death data were plotted as a function of the log of drug concentration and fitted on a ‘log(inhibitor) vs. normalized response-variable slope’ sigmoidal regression model (GraphPad prism 8.3.0). The observation was made in biological replicates and each plate of biological replicate had 6 technical repeats which were averaged for calculating percentage absorbance.

### 2.19. In Vitro Dose Response Matrix and Synergistic Effect

A total of 10,000 AsPC-1 cells per well were seeded into a 96-well flat bottom plate and incubated overnight in a humidified incubator at 37 °C with 5% CO_2_. The wells were treated with increasing concentrations of indicated drugs (0–3 µM GSM-192, 0–100 µM Gemcitabine, 0–67 µM Oxaliplatin) and allowed to incubate for 72 h. MTT solutions (M5655 SIGMA) were added at 10% of total culture volume (0.5 mg/mL MTT in RPMI/IMDM without phenol red) and after 3 h incubation, the plate was washed with PBS and solubilized using MTT solvent (0.1 N HCl in anhydrous isopropanol). The read out was obtained at 650 nm wavelength in the spectrophotometer plate reader, and the background read out was performed at 570 nm. The proliferation of the cells in the different treatment groups as a percentage of untreated control wells was calculated. Viability data were used to fit a four-parameter logistic regression (LL4) model followed by automatic outlier detection. The degree of combination synergy was quantified by comparing the observed drug combination response to the expected response, calculated using highest single agent (HSA) reference model in SynergyFinder 2.0 stand-alone web-application for interactive analysis and visualization of multi-drug combination profiling data [44].

### 2.20. Statistics

For all statistics, *n* = 3 values from independent experiments were used to calculate the s.e.m. For all representative results, experiments were repeated at least twice. Normally distributed data were tested by Student’s *t*-tests with unequal variances to compare continuous variables between two groups. Statistical analysis was performed using GraphPad Prism 8.

## 3. Results

### 3.1. Alternating Antigen Immunization Strategy Yields High Affinity Monoclonal Antibody (mAb) Selectively Targeting Active MMP-7

Previously, our lab developed an immunization strategy in which we immunized mice with a synthetic molecule (Zn Tripod) that mimicked the conserved structure of the MMPs’ catalytic zinc-histidine complex, residing within the enzyme active site [4]. This immunization procedure yielded selective function-blocking monoclonal antibodies directed against MMP-9/2 (gelatinases). Yet, this approach was found to be limited for the production of antibodies, targeting only MMP-9/2, which was endogenously present in its activated form. GSM-192 was generated using alternating antigen immunization of mice with the Zn Tripod and recombinant human MMP-7 (hMMP-7) active enzyme.

**Figure 1:**
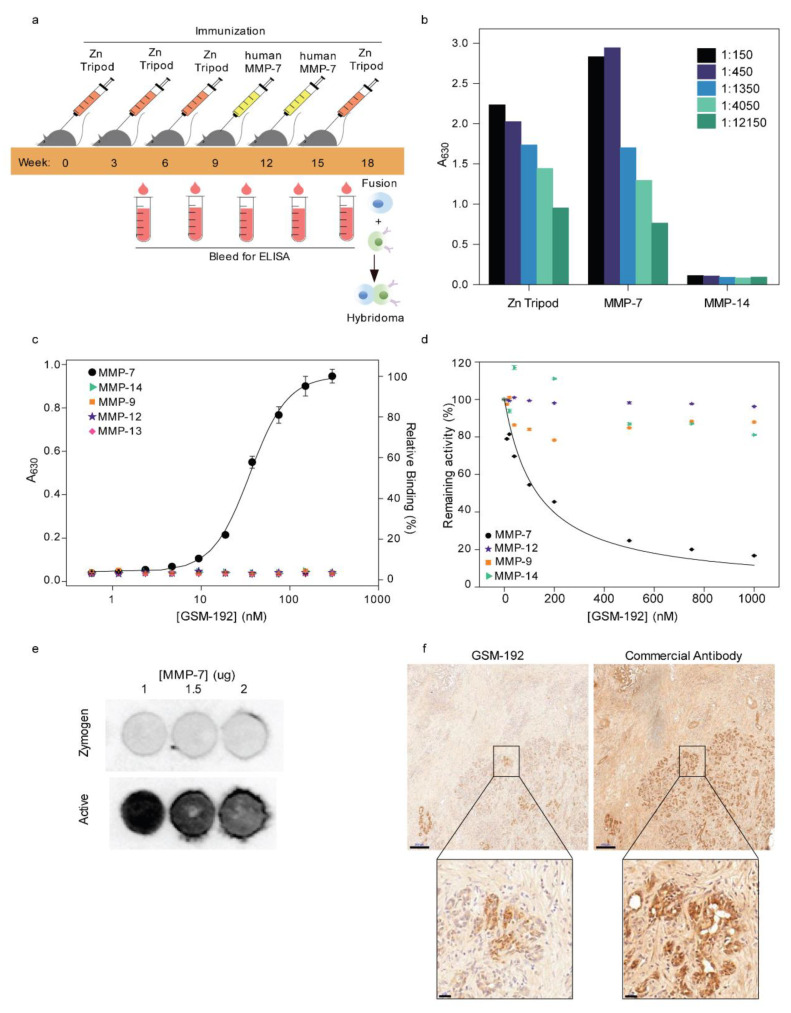
Sequential immunization yields anti MMP-7 antibody with high binding affinity and selectivity. (**a**) Female BALB/c mice were immunized in 3-week intervals with synthetic Zinc-Tripod-KLH (Zn Tripod) emulsified with complete Freund’s adjuvant or recombinant human MMP-7. Progressive immune responses were observed as a function of repetitive injection using an ELISA. B-cells from candidates with high binding affinity were selected for hybridoma generation at week 18. (**b**) Serum immune response of selected mouse prior to fusion against Zn-Tripod, MMP-7 and negative control MMP-14, showed bi-specificity towards both Zn Tripod and MMP-7 when examined using ELISA, and exhibited negligible binding with MMP-14 at various dilutions. (**c**) Purified hybridoma monoclonal antibody GSM-192 binding curve with MMP-7 active enzyme (EC_50_ = 43 ± 1 nM). The antibody did not bind effectively to a panel of other MMPs indicating its high specificity towards MMP-7. (**d**) The effect of GSM-192 Fab on the enzymatic activity of human MMP-7 was examined in vitro, using short fluorogenic peptides. GSM-192 Fab inhibited MMP-7 activity at IC_50_ = 132 ± 10 nM. Related MMPs such as MMP-14, -9 and -12 showed continued activity with negligible or no effect in the presence of GSM-192. (**e**) Dot blot showing selective binding of the anti MMP-7 Ab to catalytic form of the enzyme with high affinity at different concentrations. GSM-192 did not bind to the zymogen form. (**f**) A comparison of the GSM-192 and anti MMP-7 commercial antibody staining shows differential localization in consecutive sections of human PDAC tissue biopsies. GSM-192 predominantly stained active enzyme surrounding cancer cells (Scale bar, 200 μm), and commercial antibody stained both zymogen and catalytic enzyme. (Inset scale bar, 20 μm).

Female BALB/c mice were immunized every 3 weeks with small synthetic Zn Tripod-KLH emulsified with complete Freund’s adjuvant and the catalytic domain of hMMP-7 (see methods). The anti-Zn Tripod, anti- MMP-7 immune responses were examined in mouse serum using direct enzyme linked immune sorbent assay (ELISA). Progressive responses were observed as a function of repetitive injection (Figure 1a). Specificity of the immune responses in mouse serum was examined using ELISA against Zn Tripod and the activated MMP-7 (Figure 1b). This analysis indicated the generation of cross-reactive antibodies recognizing both Zn Tripod and MMP-7. As expected, immunization with Zn Tripod alone did not result in anti-MMP-7 antibodies (Figure S1). Next, candidate antibodies with high binding affinity to active form of MMP-7 were selected for fusion and mAb generation.

These results confirm that an immunization strategy involving alternate immunization with the synthetic mimicry molecule Zn Tripod and active forms of MMPs generates potent, highly specific mAbs with high affinity towards the target enzyme.

### 3.2. GSM-192 Selectively Binds to the Active form of Human MMP-7 and Inhibits its Catalytic Activity

The binding constant of the selected anti MMP-7 mAb GSM-192 was measured using ELISA (Figure 1c). The EC50 was found to be 43 ± 1 nM, and the antibody did not show cross- reactivity to recombinant human MMP-9, -14, -12 and -13. The lack of binding to a panel of other MMPs helped measure the novel antibody’s affinity to epitopes specifically unique to MMP-7. Interestingly, in spite of the fact that immunization booster also included the common zinc motif, the antibody was fully selective for human and mouse MMP-7.

The effect of GSM-192 Fab fragment on the enzymatic activity of human MMP-7 was examined in vitro using a short fluorogenic peptide according to a standard protocol [22]. The enzymatic activity of recombinant catalytic human MMP-7 was measured in the presence of increasing concentrations of the Fab fragment of GSM-192. GSM-192 Fab was found to inhibit MMP-7 activity with an IC50 of 132 ± 10 nM. Remarkably, the catalytic activities of MMP-9, -14 and -12 in vitro were either not affected, or, in the case of MMP-14 only negligibly affected by the anti MMP-7 Fab treatment (Figure 1d).

To test whether GSM-192 was binding to the zymogen or the active form, a dot blot experiment was conducted with varying concentrations of activated MMP-7 and its zymogen. GSM-192 bound to the active form of the enzyme, and not to the zymogen form (Figure 1e and Figure S2). Thus, our improved immunization strategy, with the introduction of active MMP-7 into the immunization process, yielded a highly selective antibody that functionally neutralized the catalytic activity of MMP-7 alone. Additionally, staining of human PDAC tissue demonstrated a selective binding of GSM-192 to the active enzyme compartment, which is a fraction of the total MMP-7 covered area stained using commercial antibody (Figure 1f).

### 3.3. Fab Fragment Crystal Structure and Docking Model Show Antibody’s Affinity to Bind Sites Close to the Conserved Active Site

**Figure 2:**
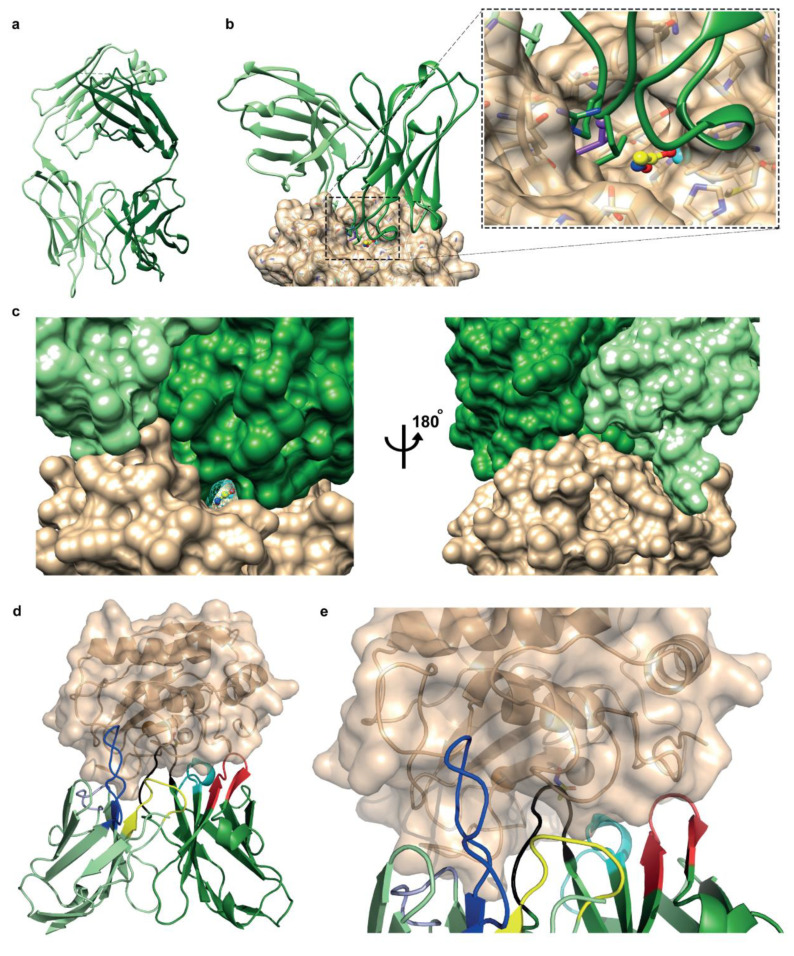
GSM-192 structure and docking showing antibody’s unique affinity to binding sites close to the conserved active site. (**a**) Crystal structure (2.3 Å) of the Fab fragment of GSM-192 represented as a ribbon diagram. Heavy chain (dark green) as well as light chain (light green) is presented (PDB: 6FBJ). (**b**) The docking model of GSM-192 to active human MMP-7 revealed no direct binding to active site. The surface of MMP-7 is marked in beige and is presented as semi-transparent. The antibody is outlined in dark green, with three side chains: L103H, which is located near the Leu anchoring spot (purple), R105H and Y33L. The latter interacts with MMP7 away from the active site. Acetohydroxamic acid (AHA) is presented as a ball-and-stick model with carbon atoms in yellow, oxygen in red and nitrogen in blue. (**c**) Docking Structure of MMP-7 (beige), GSM-192 (green) complex showing surface complementarity and a small “tunnel” through which the small inhibitor AHA can be inserted at the interface and bind to the Zn^2+^ ion. (**d**,**e**) GSM-192 antigen binding surface with complementarity determining regions. hMMP7–beige, Fab fragments–green (Heavy chain- dark green, light chain-light green), CDR3L–yellow, CDR2L–grey, CDR1L–blue, CDR3H–black, CDR2H–red, CDR1H–cyan.

To structurally understand the mAb recognition of active MMP-7, we characterized GSM-192 Fab using X-ray crystallography. The crystal structure was determined at a 2.3 Å resolution (Figure 2a). The GSM-192 Fab construct is composed of 224 amino acid residues in the heavy chain and 215 amino acids in the light chain (439 residues in total) (Data S1). The final model includes residues 1–221 in the heavy chain, and 2–185 in the light chain (Table S1). The antigen-binding surface is convoluted, and exhibits two protruding elements, one consisting of light chain CDR1 (residues 31-DSYGN-35L) and the other of the heavy chain CDR3 (residues 101-GLRR-105H); there is only a small central cavity delimited by residues N33H, H50H, F59H, R105H, D99L, and Y101L.

The docking of GSM-192 (Fv domains) to MMP-7 produced a model (Figure 2b) in which the antibody loop CDR-H3 binds inside the active site of the enzyme. The Protein Data Bank (PDB) includes several structures of the catalytic domain of MMP-7, which, when overlaid, exhibit mobility of the loops near the active site. Similar mobility is observed in normal modes analysis, and therefore the antibody GSM-192 (Fv domains L2-L112 and Q3-V119) was docked to several MMP-7 conformers that display different opening of the active site. Docking to MMP-7 conformer with an open active site, as observed in structure 2y6c [24], produced the statistically outstanding model with a complementarity score > 3σ above the next model, presented in Figure 2b.

The docking model implies that the binding is stabilized by strong hydrophobic anchoring of L103H in a pocket within MMP-7 active site, delimited by amino acids L181, A216 and Y241. Computational anchoring spots [37] mapping identified this pocket as a strong binder of hydrophobic residues, particularly Leu (Figure 2b). The neighboring residue R104H points to a negatively charged surface region, but interacts directly only with T180. Another strong hydrophobic interaction is the binding of Y33L in an elongated cavity on the surface of MMP-7 at the edge of the active site pocket, contacting residues P246 and Q247. This residue is predicted to change conformation upon MMP7 binding. Additional predicted interactions are R105H-N243, T28H-Y172, Y32H-T180, N33H-P239/T240, N52H-H229, N55H-S101, F37L-Y241, and E60L-N179. MMP7 residues T240 and Y238 point into the small cavity in the antigen binding surface. Notably, the model shows that acetohydroxamic acid (AHA), a reversible zinc binding hydroxamate that binds directly in the catalytic cleft, can be accommodated in the binding site together with the antibody. Figure 2c presents a view of the predicted structure of the MMP-7/GSM-192 complex, highlighting the surface complementarity on the one hand, and the small opening through which AHA can insert and bind to MMP-7 in the presence of GSM-192, on the other hand. In summary, the proposed binding mode of the antibody consists of hydrophobic anchoring and numerous other contacts to residues in the catalytic cleft region and its vicinity (Figure 2d,e); it does not involve Zn^2+^ and allows simultaneous binding of AHA to Zn^2+^.

The docking analysis indicates that GSM-192 interacts with hMMP-7 via a mix of highly conserved residues within the catalytic cleft; some of these are unique to MMP-7. These unique residues called specificity-determining positions (SDPs), are positioned in the vicinity of the catalytic cleft [45]. SDPs are known to be conserved between enzyme orthologs but not in enzyme paralogs [46,47]. We constructed a multiple sequence alignment (MSA) of the catalytic domains of MMP paralogs and orthologs to test whether the epitopes interacting with GSM-192 are conserved in orthologs but not in paralogs. Consequently, we identified few highly conserved positions, which constitute GSM-192 epitopes on both hMMP-7 and mMMP-7, such as H229, which interacts with N52H, and residues L181, Y241, which are key to strong hydrophobic anchoring of L103H. In addition to these highly conserved positions, we also identified partially conserved positions that are suspected to be SDPs. These positions include S101, T180, T240 and Y238. They contribute significantly towards GSM-192 selectivity to MMP-7 homologs. Altogether, the aforementioned combination of residues constitutes unique MMP-7 epitopes and results in high potency and selectivity towards both mouse and human MMP-7 orthologs.

Additionally, we superimposed the structures of mouse and human orthologs of MMP-7 to the closest human paralog with an available published structure (MMP-3). Superposition of MMP-7 human and mouse ortholologs matched (Figure S3a), while the catalytic cleft of MMP-3 did not (Figure S3b). Since this catalytic cleft region is important for antigen recognition by GSM-192 through CDR1L and CDR3H, the absence of a match will impair the interaction of GSM-192 with other members of the MMP family (Figure S3c,d).

### 3.4. Anti MMP-7 Fab Inhibits Fas Ligand Shedding Leading to Pancreatic Tumor Cell Death via Apoptosis

**Figure 3:**
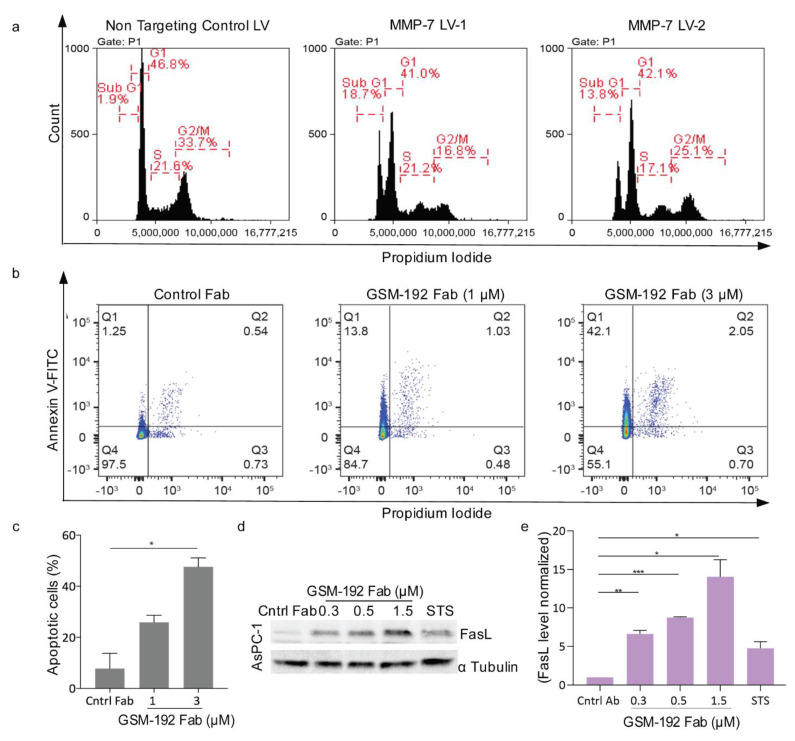
MMP-7 inhibition leads to cancer cell death in vitro. (**a**) MMP-7 lentiviral silencing resulted in increased subG1 peak indicating a leap in AsPC-1 cell death, in a FACS based cell cycle analysis. Cells exposed to MMP-7 targeting lentiviral vector (LV) showed an increase in sub G1 peak. See also Figure S4b. (**b**) MMP-7 inhibition leads to tumor cell death via apoptosis in AsPC-1 cells. FACS analysis showed increased Annexin V-related apoptosis (Q1+Q2) after treatment with increasing doses of GSM-192 Fab. FACS data output indicates early apoptotic cells as Q1, and late apoptotic cells as Q2. (**c**) Increase in percentage of cells undergoing apoptosis was plotted using mean values ± s.e.m (grey bar plot), and significance was evaluated with a two-tailed *t*-test. * *p* ≤ 0.05. (**d**) Western Blot showing FasL expression in cell lysates with and without GSM-192 treatment. An overall stable increase in FasL levels in GSM-192 treated cells was observed. Isotype control mAb at 1.5 μM concentration and staurosporine (STS), a pro apoptotic drug, were used as treatment controls. α Tubulin was used as loading control. (**e**) Data in the graph represent α Tubulin normalized mean values which were further normalized to the control mAb as baseline ± s.e.m, and significance was evaluated with a two-tailed *t*-test. * *p* ≤ 0.05, ** *p* ≤ 0.01, *** *p* ≤ 0.001.

Western blotting was used to establish the presence of MMP-7 in pancreatic cancer cell lines AsPC-1, Su86.86, BxPC-3, and CFPAC-1. Cell lysates were blotted on membranes and probed with anti MMP-7 commercial antibody. The protein levels of catalytic MMP-7, the zymogen and the intermediate form [48,49] were found to vary in the cell lines (Figures S4a and S5). CFPAC-1 and AsPC-1 had high expression of all MMP-7 forms. We tested whether the ablation of MMP-7 can affect the survival of the mono-cultured pancreatic cancer cells and carried out MMP-7 lentiviral silencing in AsPC-1 cells (Figures S4b and S5). The silencing resulted in cells with depleted MMP-7 in supernatant. They showed an augmented subG1 peak in propidium iodide (PI) staining and subsequent FACS analysis, indicating increased cell death compared to non-targeting control (Figure 3a). Thus, pancreatic cancer cells were sensitive to ablation of MMP-7.

Similarly, GSM-192-mediated MMP-7 inhibition on pancreatic cancer cells led to induction of cell death. CFPAC-1 and AsPC-1 PDAC cells died at an IC50 of 4.3 μM and 2.3 μM, respectively (Figure S4c). In this experiment, a monoclonal antibody against LOXL-2 (Lysyl Oxidase like 2) [28], was used as an isotype control. The GSM-192 Fab was shown to induce cell death via apoptosis and proportion of the cell population undergoing apoptosis increased corresponding to the increasing concentrations of anti MMP-7 treatment (Figure 3b).

**Figure 4:**
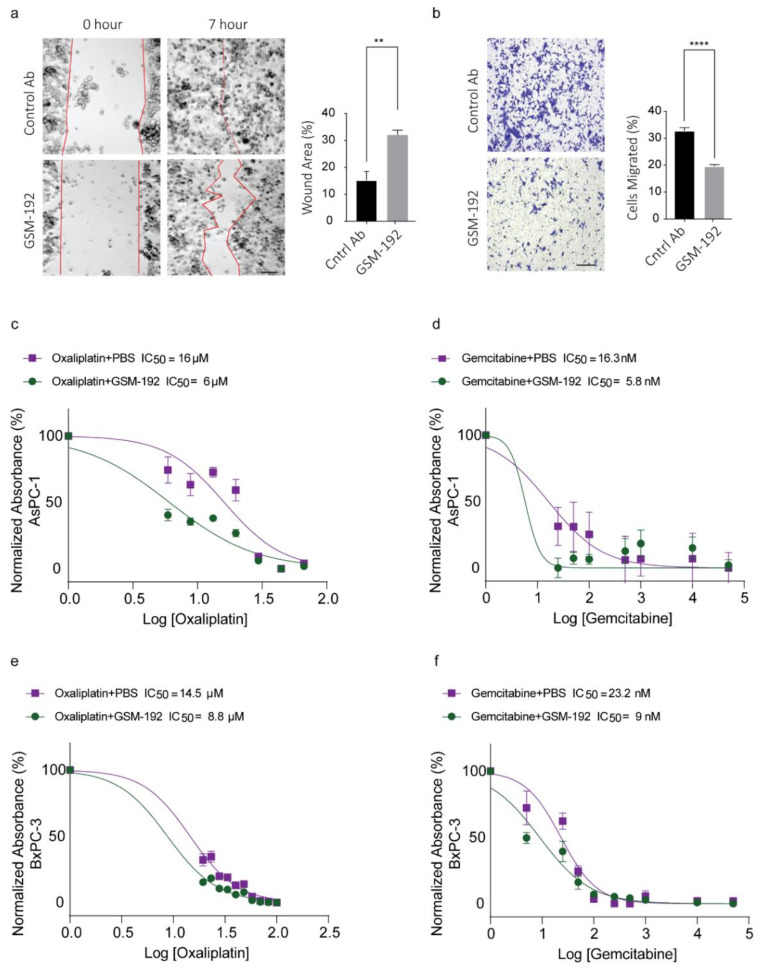
GSM-192 treatment reduces and retards cell migration and potentiates cells to chemotherapy. (**a**) Treatment with GSM-192 slowed down cell migration in a standard scratch assay. Six hours post treatment; control antibody treated wells close the scratch/wound, but not the GSM-192 treated wells. Data represent mean values ± s.e.m, and significance was evaluated with a two-tailed *t*-test. ** *p* ≤ 0.01, Scale bar, 20 μm. (**b**) GSM-192 reduces cells migrating across the transwell membrane significantly 15 h post treatment. Data represent mean values ± s.e.m., and significance was evaluated with a two-tailed t-test. **** *p* ≤ 0.0001 Scale bar, 20 μm. (**c**–**f**) MTT cell death assay absorbance data plotted as a function of the log of Gemcitabine (GEM) concentration and fitted on a variable slope sigmoidal regression model show markedly reduced IC_50_ of GEM/OX+GSM-192 group compared to the GEM/OX+PBS group in AsPC-1 (**c**,**d**) and BxPC-3 (**e**,**f**). Data from one out of three representative experiments are presented.

To test for molecular markers of apoptosis, we determined Fas ligand expression by Western blot analysis. AsPC-1 cells were treated with GSM-192 Fab for 24 h at concentrations 0.3, 0.5 and 1.5 µM. Treated cells showed an increase in Fas ligand (Fas-L) protein expression in Western blot compared to control (Figure 4c and Figure S2). In sum, ablation of MMP7 through GSM-192 in pancreatic cancer cell lines stabilized FasL expression and induced apoptosis, and reduced viability.

### 3.5. GSM-192 Fab Reduces Cell Motility In Vitro

MMP-7 mediated E-Cadherin cleavage promotes cell migration [16], so we hypothesized that an effective anti MMP-7 antibody directed to the catalytic enzyme should be able to impact migration. In a standard scratch assay (Figure 4a) 1 µM GSM-192 treatment showed significant reduction in rate of wound closure (as controlled for anti-proliferative effect using Mitomycin C on CFPAC-1 cells in a prior experiment, data not shown). Furthermore, treatment with 1 µM GSM-192 reduced the number of cells able to migrate across the transwell membrane 15 h post treatment almost by half (Figure 4b).

### 3.6. Co-Treatment with GSM-192 Decreases IC_50_ of Gemcitabine (GEM) and Oxaliplatin (OX)

Thus far, we have shown that treatment of cell cultures with GSM-192 mAb or Fab affects critical properties of cancer cells, such as viability and motility. In addition, it was shown that MMP-7 is implicated in acquiring drug resistance via dysregulation of Wnt/β-catenin pathway [50] and shedding of Fas [21]. Consequently, we checked the effectiveness of GSM-192 to overcome MMP-7 mediated drug resistance in PDAC cell lines AsPC-1 and BxPC-3. Such improvement is highly desirable since pancreatic ductal adenocarcinoma is notoriously resistant to chemotherapeutic agents. Our treatment of AsPC-1 cells yielded an IC_50_ of 13.3 ± 3.9 µM in OX group and an IC_50_ of 15.4 ± 1.3 nM in GEM group. Co-treatment with GSM-192 showed a reduction in the IC_50_ of OX to 6.2 ± 0.2 µM and IC_50_ of GEM to 6.6 ± 1.2 nM (Figure 4c,d). Similarly, in BxPC-3 cells co-treatment reduced the IC_50_ of OX to 8.6 ± 0.2 µM and GEM to 7.3 ± 2.3 nM from 13.3 ± 1.8 µM and 18.4 ± 6.9 nM, respectively (Figure 4e,f).

The synergy scores were calculated using HSA reference model for GSM-192 with GEM and OX co- treatment in AsPC-1 cells using SynergyFinder tool [44]. The most synergistic area scores were 13.90 and 13.34 for GEM and OX, respectively (Figure S6a–d). Score above 10 indicates that the interaction between two drugs is likely to be synergistic. Therefore, anti MMP-7 mAb GSM-192 could be effectively used to reduce resistance to chemotherapy and reduce drug toxicity by dose optimization.

Although GEM alone is known to upregulate FasL in non-small cell lung cancer (NSCLC) [51], 200 µM Gem treatment alone of hPDAC AsPC-1 cells did not result in noteworthy increase in FasL levels in the cell fraction. However, GSM-192 treatment as observed earlier did result in a concentration dependent increase in FasL levels in cells undergoing co-treatment (Figures S5 and S7).

## 4. Discussion

Blocking abnormal MMP enzyme activity in its remodeling capacity and as a cell surface shedder is desirable in treating a number of diseases including cancer. An increasing body of literature points to MMP-7 as a particularly interesting MMP drug target in need of specific inhibitor and target validation, especially in cancer. To target MMP-7 with high affinity, we developed a conformational selective antibody using a unique immunization strategy alternating between Zn Tripod and recombinant human MMP-7. Presenting the recombinant active form of MMP-7 as a secondary antigen promoted the generation of cross-reactive antibodies recognizing both Zn Tripod and MMP-7. Importantly, the isolated clone GSM-192 exhibited impressive specificity and affinity to MMP-7 when compared to closely related MMPs. Docking of the Fv domains of the antibody crystal structure to available MMP-7 structures suggested GSM-192 to be effective in binding the catalytic cleft region, producing a stearic hindrance blocking entry of substrate molecules.

Our initial experiments with lentiviral silencing of MMP-7 in vitro in PDAC cells indicated the importance of MMP-7 in pancreatic cancer cell viability. Pancreatic cancer is a lethal and highly aggressive malignancy [52]. While its incidence is dramatically increasing, the treatment options for pancreatic cancer remain scarce [53], and novel therapeutic strategies are urgently needed [54]. In pancreatic cancer, serum MMP-7 was recently shown to be a pre-operative prognostic marker, as its increased expression correlated with unresectable disease [55]. Previous evidence indicated that MMP-7 in serum [56], plasma, and pancreatic juice [57], along with related disease indicators, could be used as a diagnostic marker. Elevated serum MMP-7 levels in human patients with PDAC correlated with metastatic disease and decreased survival [6].

Hence, diagnostic tools could benefit from utilizing GSM-192 to monitor plasma derived MMP-7 as a biomarker, or as an antibody conjugated to fluorescent tags or nanoparticles to track activated enzymes in vivo (utilizing non-invasive imaging technology). Tissue staining for PDAC may be improved by specifically staining for the active enzyme compartment using GSM-192 (Figure 1f).

MMP-7 has been shown to accord protection to tumor cells from apoptosis via stabilization of major extrinsic death pathway receptor and ligand duo Fas [13], as well as Fas-L [58]. Inhibition of MMP-7 using GSM-192 increased apoptotic cell death, along with a concentration dependent stabilization of the Fas ligand. Furthermore, GSM-192-treatment of PDAC cells (CFPAC-1) impaired their ability to migrate through transwell pores, and close an artificial wound in a scratch assay. The role of MMP-7 in promoting cell migration via E-Cadherin ectodomain shedding is well-established [16], and our data corroborate the pro invasive role played by MMP-7 in PDAC.

There are limitations in using GSM-192 as a single agent in diseases such as PDAC with a complex extracellular protease web with enzymes characterized by redundant activity profiles and compensatory effects in vivo. Alternatively, GSM-192 could be effective in a combinatorial drug cocktail with clinically proven chemotherapeutics. High MMP-7 level is an independent predictor of docetaxel resistance and poor cancer survival in patients with castration-resistant prostate cancer (CRPC) [59]. Enhanced drug efflux activity was found to be correlated with induced expression and activity of MMP-7 in a HSP-90 dependent manner [60]. Activation of MMP-7 by DKK1 and Wnt/β-catenin pathway is known to induce T-DM1 or ado-trastuzumab resistance, and lead to poor prognosis in gastric adenocarcinoma [61].

**Figure 5:**
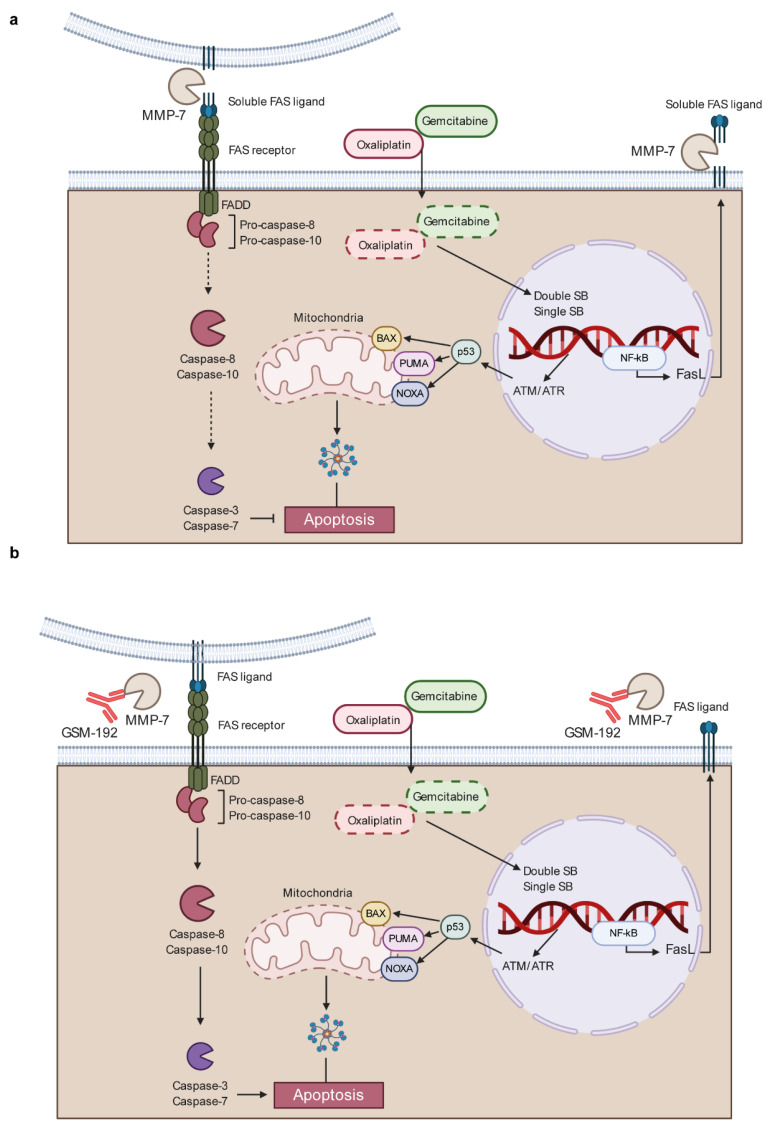
Schematic representation of the link between chemoresistance and MMP-7 shedding of Fas ligand. (**a**) DNA chelating chemotherapy agents including oxaliplatin and gemcitabine create double and single strand breaks activating DNA repair ATM/ATR signaling pathways. Failure of these repair pathways lead to p53 mediated activation of an intrinsic death pathway and upregulation of cell surface FasL expression. Cancer cells overcome increased extrinsic death signaling by increasing MMP-7 expression leading to FasL shedding. (**b**) GSM-192 mAb neutralizes MMPs enzyme activity, restraints chemo resistance by preventing Fas ligand shedding and restore the extrinsic pathway of death signaling.

Commonly used chemotherapeutic drug regimens in clinic for PDAC include a combination drug regimen called FOLFIRINOX (folinic acid, fluorouracil, irinotecan, and oxaliplatin), gemcitabine [62,63], or gemcitabine with nab-paclitaxel [64]. Mindful of the fact that lowering drug concentration is beneficial, we tested the ability of GSM-192 to sensitize AsPC-1 and BxPC-3 PDAC cells to gemcitabine and oxaliplatin treatment. GSM-192 treatment considerably enhanced drug efficacy, indicated by lowering of IC_50_, leading to a substantially lower drug concentration required to achieve the desired cell kill. This combinatorial effect of GSM-192 with OX or GEM was synergistic at certain concentrations, or at the very least additive in all other concentration combinations. Gemcitabine and oxaliplatin are known to activate intrinsic cell death mechanisms, following DNA damage, and cancer cells are known to upregulate MMP-7 to attenuate the Fas/FasL based extrinsic pathway for apoptosis [65] resulting in increased chemoresistance and evasion of death signaling. The enhanced cell kill due to drug synergy coincided with concomitant increase in FasL levels in the cell fraction. We thus propose that the restoration of the Fas based extrinsic pathway in addition to the extant drug induced cell death driven by the intrinsic death pathway results in a more effective cell kill. GSM-192 could be effective in countering, not only the inherent resistance to Fas and FasL based cell death in PDAC [66], but also the chemotherapy induced enhancement of MMP-7 expression and FasL shedding (Figure 5). Further, we recognize the importance of using in vivo murine genomic PDAC models such as the KPC [67] to further test the beneficial effects of GSM-192 treatment.

Previous attempts to block matrix protease activity failed to attain the desired results, due to lack of selectivity of broad spectrum small molecule inhibitors, off-target interactions, poorly conceived pre-clinical or clinical trials and the complexity of the protease web coupled with limited understanding of the same [68,69]. Targeting only the active conformation of a MMP with a protein based inhibitor, which exhibit high selectivity owing to its protein-protein interactions with both highly conserved sites as well as unique specificity-determining positions, could be key to tackling these challenges. GSM-192 mAb is one such unique next generation inhibitor, capable of selectively inhibiting MMP-7 active enzyme with high affinity. GSM-192 may be used as a monotherapy or in combinatorial drug regimen in specific fibro-inflammatory disease stages with deleterious MMP-7 enzyme activity.

## 5. Conclusions

MMP-7 sheds cell surface proteins, amplifying its role in diseases including cancer. We provide antibody-based conformation selective inhibition as a novel tool to explore the mechanistic roles of elevated MMP-7 activity. The development of GSM-192 may have far-reaching implications for studying pathologies with elevated MMP-7 including PDAC. GSM-192 is useful as a diagnostic tool, precisely tracking MMP-7 activity. Moreover, our results suggest that GSM-192 could be effective in a combinatorial drug cocktail with clinically proven chemotherapeutics.

## Data Availability

All data generated or analyzed during this study are included in this article (and its Supplementary Information files). GSM-192 X-ray structure was deposited in the protein data bank with accession number 6FBJ.

## Supplementary Materials

The following are available online at https://www.mdpi.com/article/10.3390/cancers13071679/s1, Figure S1: ELISA showing lack of anti MMP-7 antibodies, in immunization with synthetic Zinc-tripod alone, Figure S2: Original unedited western and dot blot images from Figure 1e and Figure 3c, Figure S3: Superimposition of structures orthologs of MMP-7 orthologs with MMP-3 a closest human paralog, Figure S4: MMP-7 expression and cell survival post GSM-192 treatment., Figure S5: Original unedited western blot images from Figures S3a,b and S5, Figure S6: Visualization of synergy scores for drug and GSM-192 combination in AsPC1 cells, Figure S7: GSM-192 treatment raises FasL levels and synergizes with chemotherapeutic drug gemcitabine for enhanced cell kill, Table S1: Data collection and crystallographic refinement statistics of GSM-192 Fab, Data S1: GSM-192 sequencing.
