# Supplementary material for: Conformation-Specific Inhibitory Anti-MMP-7 Monoclonal Antibody Sensitizes Pancreatic Ductal Adenocarcinoma Cells to Chemotherapeutic Cell Kill
Source: Cancers (Basel). 2021 Apr 2;13(7):1679. doi: 10.3390/cancers13071679 (PMC8038143; doi:10.3390/cancers13071679)
Supplement: Supplementary file 1 [file cancers-13-01679-s001.pdf]

Conformation-specific inhibitory anti-MMP-7 monoclonal antibody sensitizes pancreatic ductal adenocarcinoma cells to chemotherapeutic cell kill

Vishnu Mohan, Jean P. Gaffney, Inna Solomonov, Maxim Levin, Mordehay Klepfish, Sophia Akbareian, Barbara Grünwald, Orly Dym, Miriam Eisenstein, Kenneth H. Yu, David P. Kelsen, Achim Krüger, Dylan R. Edwards, Irit Sagi

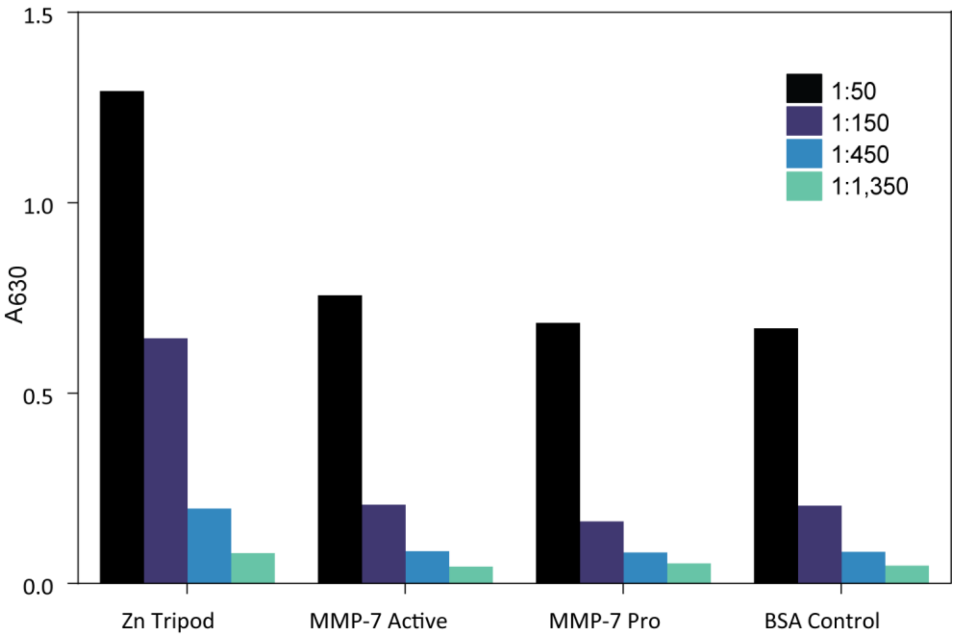

Figure S1. ELISA showing lack of anti MMP-7 antibodies**, in immunization with synthetic Zinc- tripod alone.** Immune responses were examined in serum using ELISA against antigens Zinc-Tripod, MMP-7 active form, MMP-7 zymogen form and BSA control in bleed from mouse immunized with Zinc-Tripod alone. The ELISA did not show presence of antibodies against MMP-7 catalytic or zymogen forms.

Figure 1e

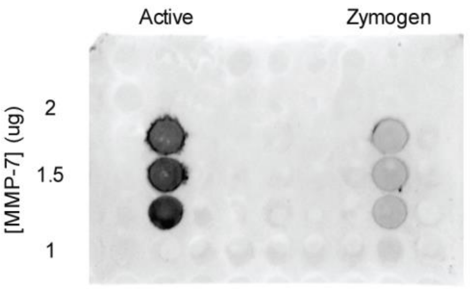

Figure 3c

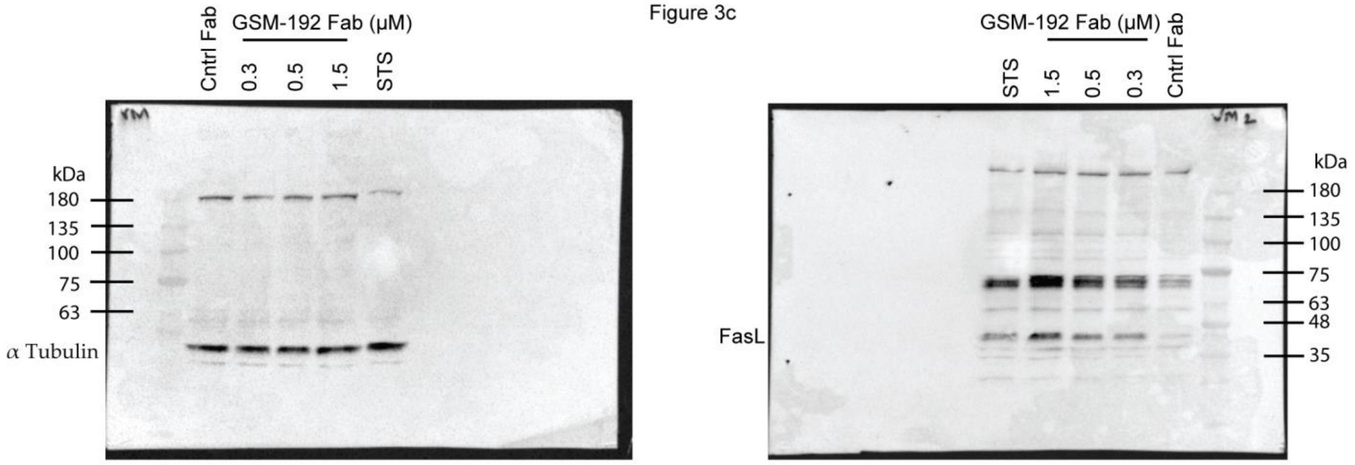

Figure S2. Original unedited western and dot blot images from Figures 1e, 3c.

.

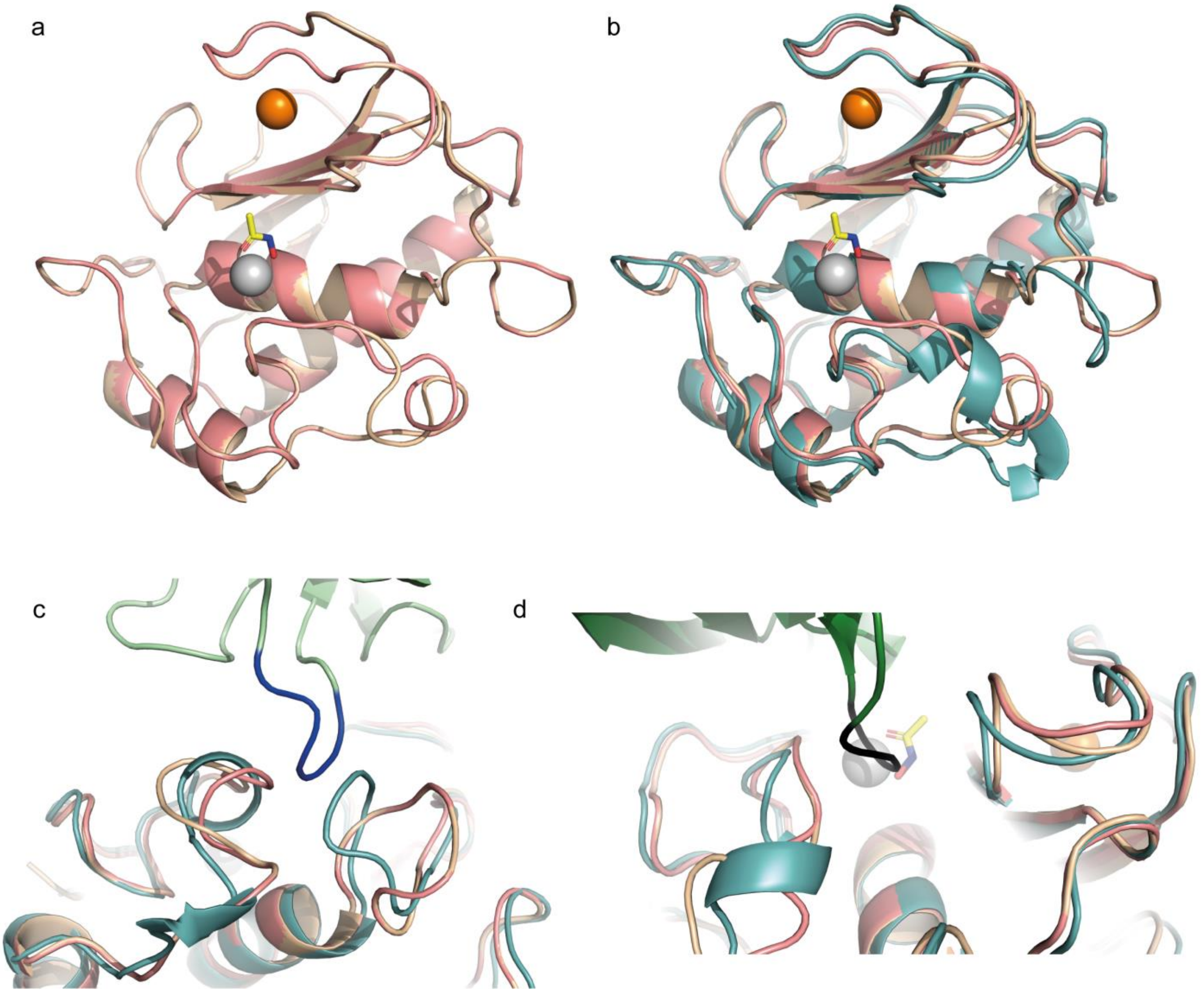

Figure S3. Superimposition of structures orthologs of MMP-7 orthologs with MMP-3 a closest human paralog. (a) The superimposition of orthologs shows a match in the catalytic cleft region. (b) The structures don't match in comparison between paralogs. hMMP7 – beige, mMMP7 – salmon, MMP3 – teal. (c,d) The catalytic cleft region is important for association with antigen recognizing CDRs CDR1L (blue) and CDR3H(black) of GSM-192.

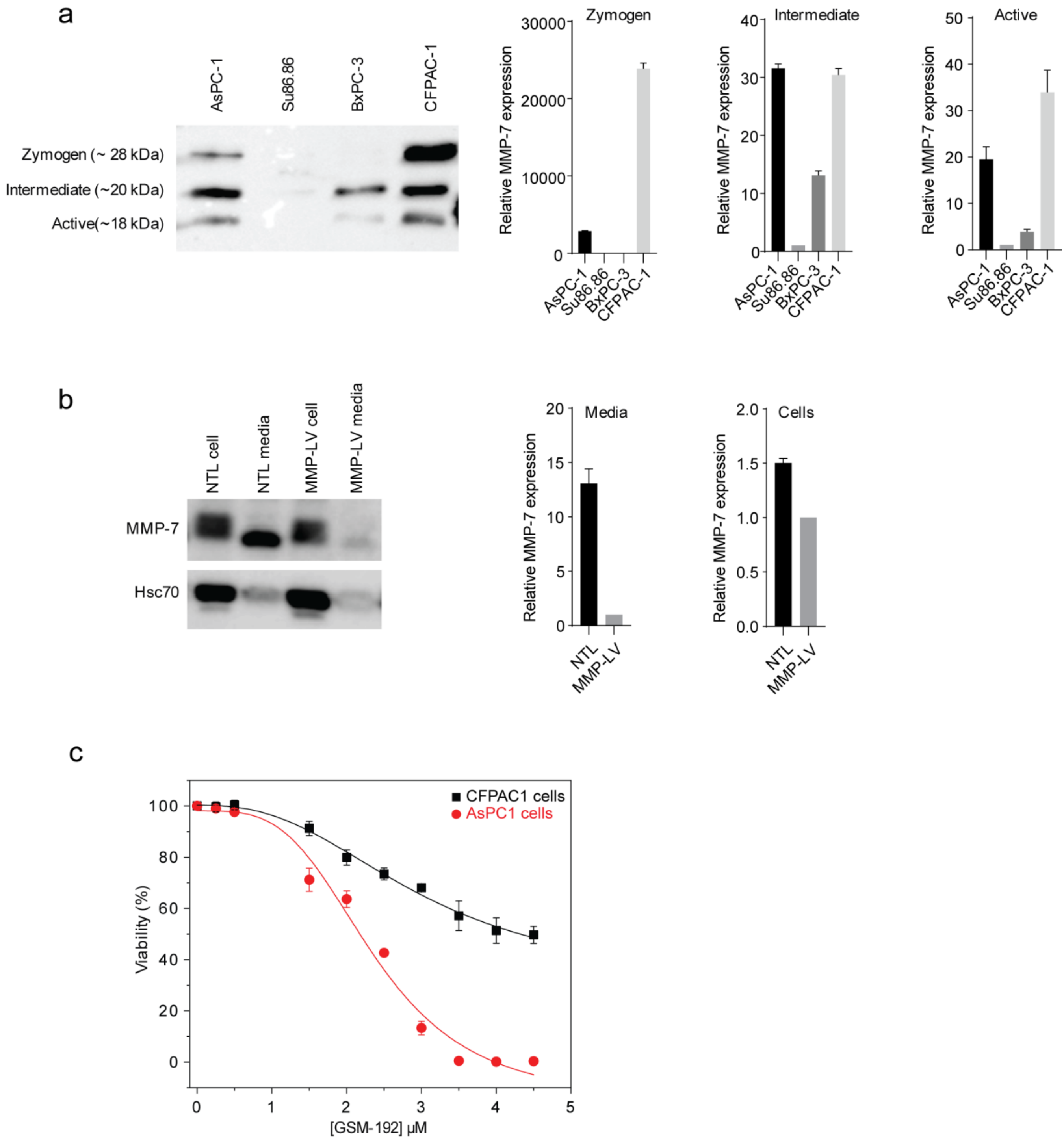

Figure S4. MMP-7 expression and cell survival post GSM-192 treatment. (a) Western blot analysis of cell culture media showing MMP-7 expression in various pancreatic cancer cell lines. Data in the graph represent relative mean densitometry values ± s.e.m. (**b**) Western blot confirming the knockdown efficacy of MMP-7 in lentiviral (LV) silencing. MMP-7 is efficiently knocked down in targeting LV when compared to non-targeting LV samples. Loading control is Hsc70. Data in the graph represent relative mean densitometry values ± s.e.m, cell fraction MMP-7 levels normalized to Hsc70. (**c**) The MTT cell survival assay was used to generate dose response curve-fitting analysis and IC_{50} values for GSM-192 treatment on AsPC-1 and CFPAC-1 pancreatic ductal adenocarcinoma cells were obtained. In this experiment a monoclonal antibody against LOXL-2 (Lysyl Oxidase like 2), was used as an isotype control. Data in the graph represent mean values ± s.e.m,.

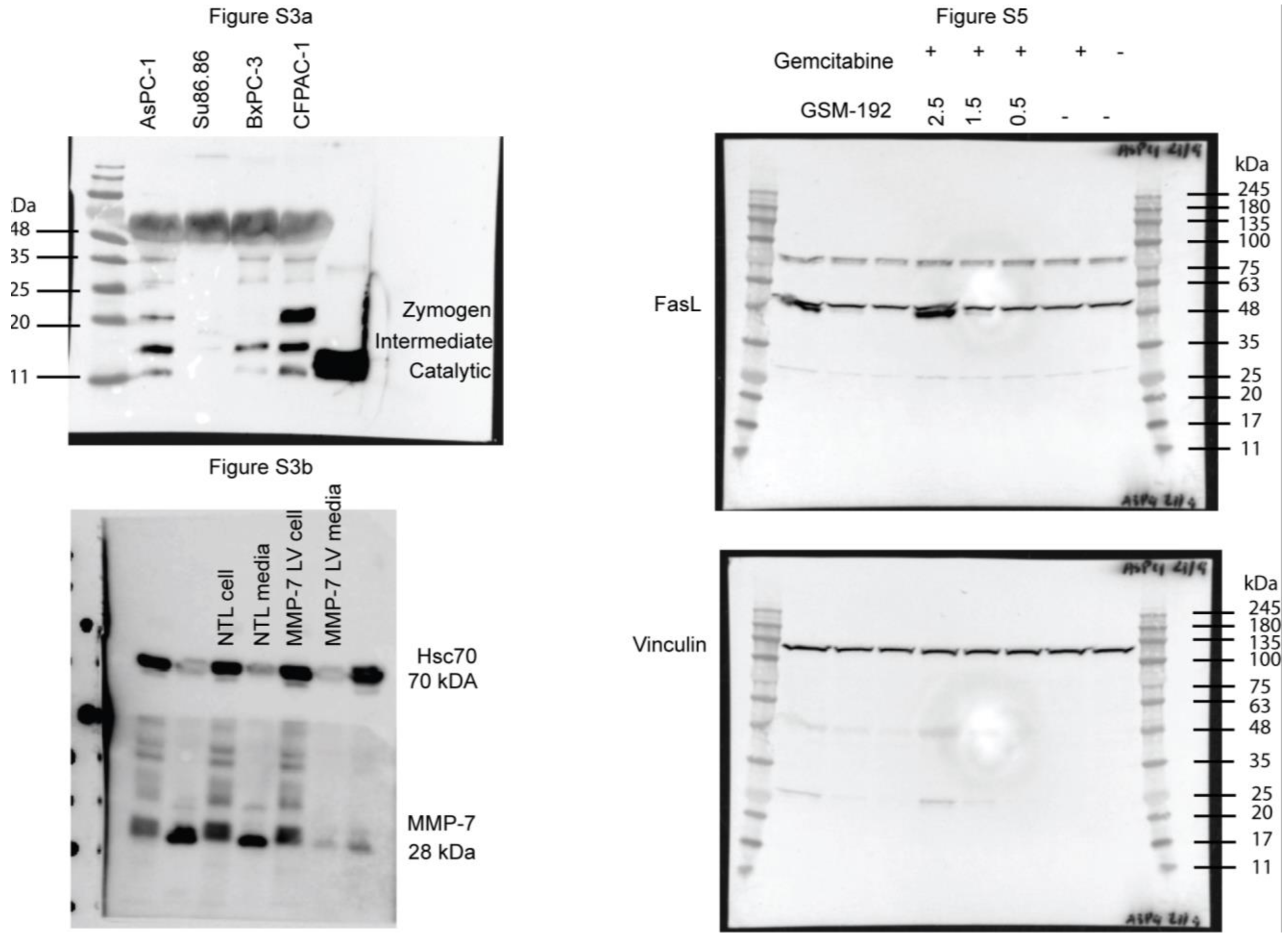

Figure S5. Original unedited western blot images from Figures S3a, S3b, S5.

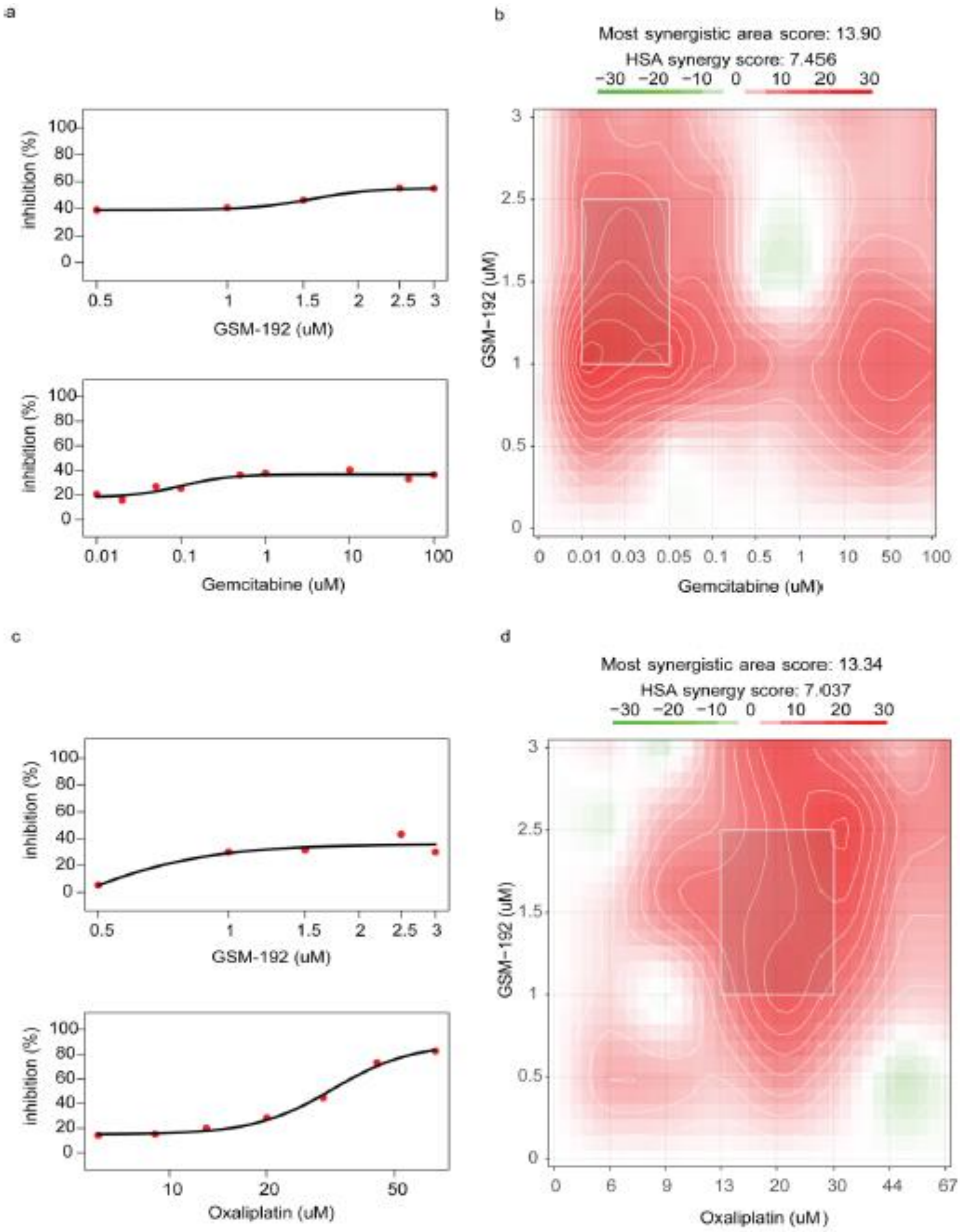

Figure S6. Visualization of synergy scores for drug and GSM-192 combination in AsPC1 cells. SynergyFinder stand-alone web-application for interactive analysis and visualization of multi-drug combination profiling data was used to generate individual dose response curve (**a**,**c**) and synergy map using highest single agent (HSA) reference model (**b**,**d**). (**a**,**b**) correspond to Gemcitabine, GSM-192 combination with a most synergistic areas score of 13.90 signifying a degree of combination synergy. (**d**,**e**) corresponds to Oxaliplatin, GSM-192 combination with a most synergistic areas score of 13.34 also signifying a degree of combination synergy.

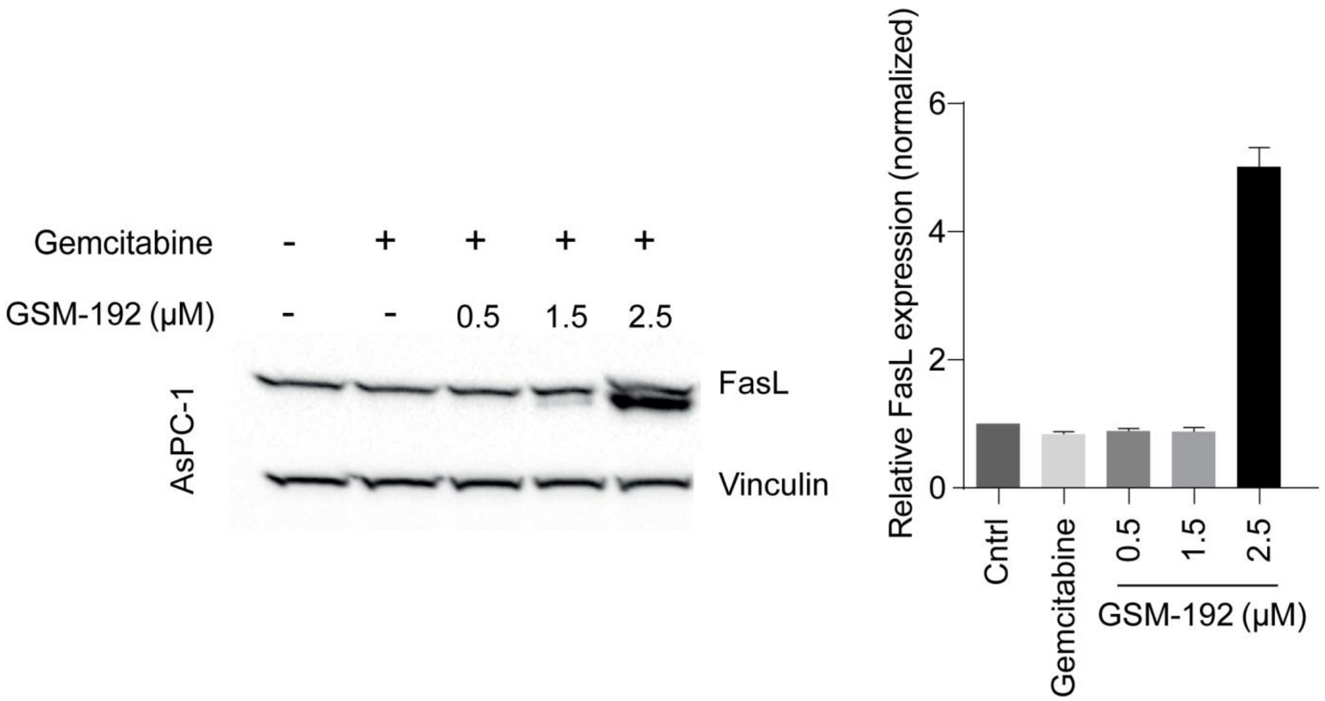

Figure S7. GSM-192 treatment raises FasL levels and synergizes with chemotherapeutic drug gemcitabine for enhanced cell kill. Western blot showing FasL levels in AsPC-1 cells. A concentration dependent increase in FasL levels in GSM-192 treated group enhances extrinsic pathway mediated cell kill in 200μm gemcitabine treated AsPC-1 cells. Gemcitabine treatment alone did not result in noticeable increase in FasL levels. Shown are representative results from 2 independent experiments. Data in the graph represent vinculin normalized relative mean densitometry values, which were further, normalized to the untreated control as baseline ± s.e.m.

Table S1. Data collection and crystallographic refinement statistics of GSM-192 Fab.

**Data collection**	
Resolution range (Å)	50.0–2.30 (2.34–2.30) [a]
Space group	$P3_121$
Unit cell dimensions	
a, b, c (Å)	88.44, 88.44, 119.34
g (°)	120
Number of molecules in the asymmetric unit	1
Number of reflections measured	870,771
Number of unique reflections	24,579(1,203) [a]
R_{sym}	0.085 (0.58) [a]
Completeness (%)	100.0 (100.0) [a]
Redundancy	12.0 (11.9) [a]
<I>/<s(I) >	33.0 (5.2)[a]
**Refinement statistics**	
Resolution (**Å**)	38.3–2.30
Rwork (%)	23.22
Rfree (%)	27.85

**B-factor (Å²)**	
Protein	37.7
**RMSD from ideal geometry**	
rmsd bond length (Å)	0.009
rmsd bond angles (°)	1.2
**Ramachandran plot (%)**	
Most favored	85.8
Additional favored	13.3
Generously allowed	0.0
Disallowed regions	0.9

[a] Values in parentheses correspond to the highest-resolution shell.

Data S1: GSM-192 sequencing.

Immunoglobulin V region genes were cloned and sequenced after amplification by PCR and following sequence was obtained-

GSM-192 Light Chain Sequence

D I VT Q S P A S L A V S L G Q R A T I S C R A S E S F D S Y G N T F V H W Y Q Q K P G Q P P K L L I Y L V S N L E S G V P A G F R G R G S R T D F T L T I D P V E A D D A A T Y Y C Q Q N N E D P Y T F G G G T K L E I K R A

GSM-192 Heavy Chain Sequence

E V Q L Q Q S G P E L V K P G A S V K I P C K A S G Y T F T D Y N M D W M K Q S H G K S L E W I G H I N P N N G G T F Y N Q K F K D K A T F I V D K S S N T A Y M E L R S L T S E D T A V Y F C A R G G G L R R G P F A Y W G Q G T L V T V S
